# Global Trends in Atherosclerosis Research in the Epigenetics Field: Bibliometric and Visualization Studies

**DOI:** 10.3390/ijerph182413154

**Published:** 2021-12-13

**Authors:** Linying Jia, Ao Cheng, Naqash Alam, Yuxuan Qian, Zeyao Ma, Honghao Ren, Rong Wang, Enqi Liu

**Affiliations:** Laboratory Animal Center, Xi’an Jiaotong University, Xi’an 710061, China; jialinying95202@stu.xjtu.edu.cn (L.J.); along999@stu.xjtu.edu.cn (A.C.); khannaqash555@gmail.com (N.A.); qyx1259860702q@stu.xjtu.edu.cn (Y.Q.); 2194120340@stu.xjtu.edu.cn (Z.M.); renhonghao@stu.xjtu.edu.cn (H.R.); rongw1986@mail.xjtu.edu.cn (R.W.)

**Keywords:** atherosclerosis, epigenetics, publication, bibliometrics, DNA methylation

## Abstract

Atherosclerosis is a pathological vascular state caused by the interaction of environmental and hereditary factors. Epigenetic modifications may be the bridge connecting environmental factors and genetic factors. A search for publications on the Web of Science database in the field of atherosclerosis related to epigenetics was conducted from the earliest mention to 31 December 2020. Data on total and annual publications, citations, impact factors, Hirsch (H)-index, citation times, most prolific authors, and frequently published journals were collected for quantitative and qualitative comparison. A total of 1848 publications related to epigenetics and atherosclerosis were found. The major contributing countries were the China (522, 28.23%), United States (485, 26.23%), and Germany (119, 6.44%). The greatest number of retrieved publications were published in the journal, “Arteriosclerosis, Thrombosis, and Vascular Biology” (62, 3.66%). The publication “Oxidative Stress and Diabetic Complications” was cited 2370 times. The most frequent keywords were “DNA methylation” and “LncRNA”. Publications on epigenetic research in the atherosclerosis field have increased significantly every year, indicating that the study of epigenetic modifications plays an increasingly important role in understanding the pathology of atherosclerosis.

## 1. Introduction

Atherosclerosis is a chronic inflammatory disease manifesting with pathological changes in the cardiovascular system caused by the interaction of environmental and genetic factors [1]. The environmentally-induced phenotype is dependent on epigenetic regulation [2], and recent studies have shown that epigenetics plays an important role in the occurrence and development of atherosclerosis [3]. Further explorations into the effects of epigenetic modifications on the formation and development of atherosclerosis are expected to further elucidate the pathogenesis of atherosclerosis [4,5,6]. The reversibility of epigenetic modifications may open up new strategies and targets for the treatment of atherosclerosis [7].

Epigenetic modifications include all types of genetic changes that modulate gene expression without involving potential changes in DNA sequence [8]. It also refers to the process by which cells can retain information about prior states and memories of disturbances without changing the DNA sequence [9] by remodeling chromatin structure to alter gene expression, through other epigenetic modifications, transcription factors, and translation mechanisms [10]. Epigenetic plays a crucial role in gene expression regulation during development, stress and disease [11]. Epigenetic mechanisms include DNA methylation [4], histone modification [12], chromatin remodeling [13], noncoding RNA modification [14], histone modification (including acetylation) [15], methylation [16], phosphorylation [17], ubiquitination [18], and glycosylation [19]. These modifications regulate gene expression by altering chromatin structure and creating binding sites for chromatin-related proteins. Many studies have suggested that the pathogenesis of atherosclerosis is related to dynamic epigenetic modification [20], but reserving atherosclerosis by influencing epigenetic modification is an urgent problem to be solved in clinical research [21]. It is necessary to provide relevant researchers with the research progress, research trends, and research hotspots in this field.

Bibliometric analysis is an important tool to evaluate the latest trends in current scientific research [22]. This technology allows people to obtain information about scientific output of individuals, institutions, and countries by using relevant parameters, including quantity, journal impact factor (IF) and citation frequency of published papers, which contribute to predicting the development of certain research fields [23]. This kind of analysis is usually used to evaluate a large number of scientific papers in a specific research field [24]. Bibliometric analysis was applied to cancer, exosome [25], coronavirus, cardiovascular disease [26] and other fields. It is recognized as a systematic analytical technology that plays a guiding role in the formulation of policies and the establishment of clinical guidelines [27].

In this manuscript, bibliometric tools were used to analyze publications related to atherosclerosis in the epigenetic field retrieved from the Web of Science (WOS) databases. Previous studies have often been presented in the form of still images, and interactive images are used in our paper to show the correlation between research contents in the field of atherosclerosis in the field of epigenetic modification. The innovation of this paper lies in the visual analysis of the research hotspots and trends related to atherosclerosis in apparent modification as well as previous research emphases, which has guiding significance for researchers involved in related fields. Over time, the research focus of atherosclerosis epigenetic modification has shifted from DNA methylation to lncRNA.

## 2. Materials and Methods

### 2.1. Data Sources

Based on the publications in the WOS database (http://www.webofknowledge.com/, accessed on 3 July 2020), we conducted a bibliometric analysis of the research results from the earliest mention in 1978 to 2020 by online bibliographic retrieval as of 31 December 2020 [28].

### 2.2. Search Strategy

All published information on the WOS was collected, and the database was validated as of 31 December 2020. In our study, the research terms were as follows: TS = atherosclerosis * AND epigenetic. In order to ensure that the publications we retrieved were comprehensive and accurate, we searched the following keywords separately: TS = atherosclerosis * AND DNA methylation, TS = atherosclerosis * AND histone modification, TS = atherosclerosis * AND chromatin remodeling, TS = atherosclerosis * AND non-coding RNA, and TS = atherosclerosis * AND histone modification. We also carried out a more detailed keyword search: TS = atherosclerosis* AND Histone Acetylation, TS = atherosclerosis* AND histone phosphorylation, TS = atherosclerosis* AND histone glycosylation, TS = atherosclerosis* AND ubiquitination of histone, and TS = atherosclerosis* AND histone methylation. Document type = (articles and reviews), year of publication = (all years), and language = English.

All the retrieved publications were filtered to remove duplicate publications. Data entries and collections were verified by two authors (Linying Jia and Ao Cheng).

### 2.3. Data Collection

The total records of papers from the WOS database, including publication year, title, authors’ names, affiliations, nationalities, abstracts and key words, journal names, etc., were saved in Txt format and opened by Excel 2019. Our collaborators, Linying Jia and Ao Cheng, browsed and extracted the data from these publications. Any disagreement was resolved through a meeting to reach a final consensus. Lastly, the extracted data were analyzed by Microsoft Excel 2019 and VOSviewer.

### 2.4. Bibliometric Analysis

From the downloaded data, Microsoft Excel 2019 (Microsoft, Albuquerque, NM, USA) extracted the number of papers published by each country, the percentage of the total number of publications, the citation frequency of publications in this field for each country, and the average citation frequency. In addition, the contribution of each institution and the major journals in the field were extracted and depicted as a histogram from the downloaded data. Scientific networks also allow analysis of many publishing characteristics, including country and region, institution, publication time, author, and cited frequency.

### 2.5. Visual Analysis

VOSviewer is a visual bibliometric network-building software developed by the Center for Science and Technology Studies (CWTS) of Leiden University in The Netherlands (https://www.vosviewer.com/ accessed on 3 July 2020). This software can extract the author, institution, abstract, country, and other related information from literature search data. These can then be used for visual analysis; mapping of scientific knowledge; and illustration of the structure, evolution, and cooperation within a specific area of knowledge [29]. The processing flow of VOSviewer is similar to other software for graphing scientific research trends: file import author, keyword, and other information extraction; building a co-occurrence matrix; using similarity calculations to standardize the relationships; and statistical analysis for accurate visual display. For the keyword map, a full counting method was used, meaning that each co-occurrence link carried the same weight. VOSviewer software extracts and analyzes the semantic content of titles, abstracts, and keywords of publications, correlating recurring keyword data and producing a visual result.

## 3. Results

### 3.1. Contributing to Atherosclerosis and Epigenetics

**Figure 1:**
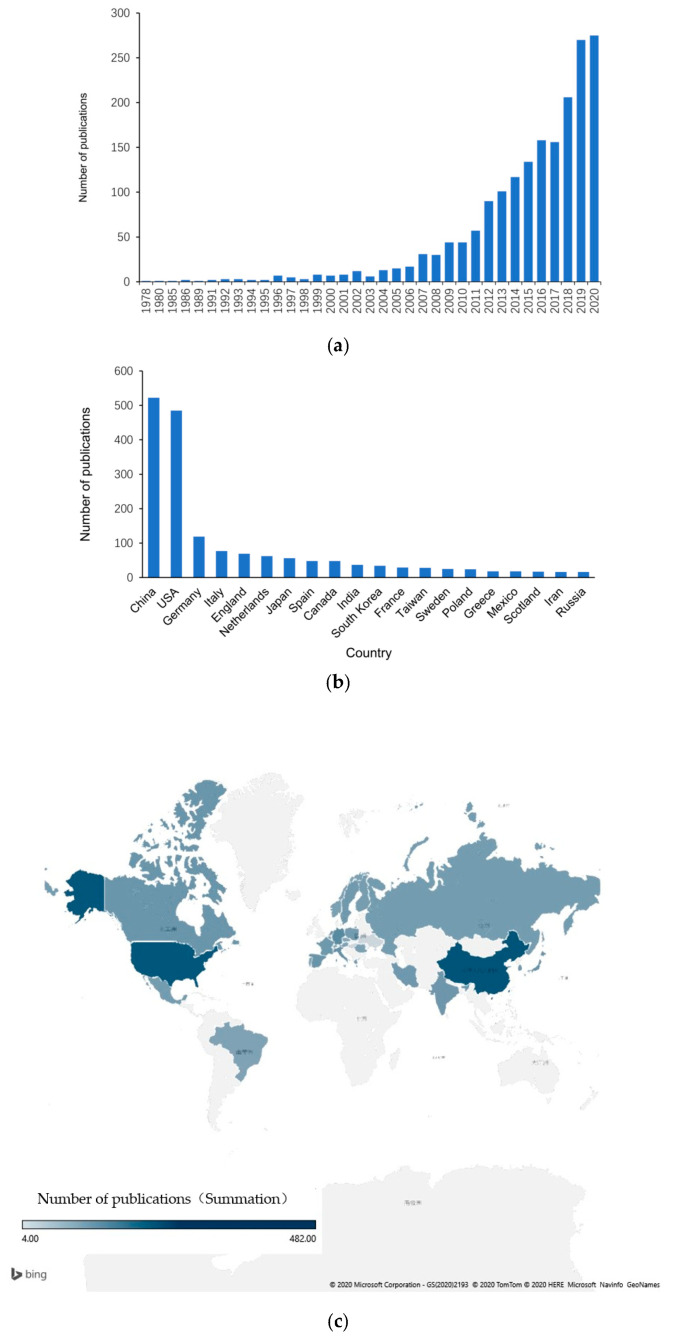
Global trends and countries contributing to atherosclerosis research. (**a**) Global number of related articles by year. (**b**) Top twenty countries with the highest number of published articles. (**c**) Global map showing countries where the articles were published.

According to survey statistics, a total of 1848 publications were collected from 1978 to 2020. In terms of number of publications per year, 2020 had the highest at 279 (15.06%), indicating that atherosclerosis is a rapidly developing research area in the epigenetics field (Figure 1a). Among the countries publishing in this area, China published the most relevant publications or reviews (522, 28.23%), followed by the United States (485, 26.23%), Germany (119, 6.44%), Italy (77, 4.16%), and England (69, 3.73%) (Figure 1b,c).

### 3.2. Citation Numbers

**Figure 2:**
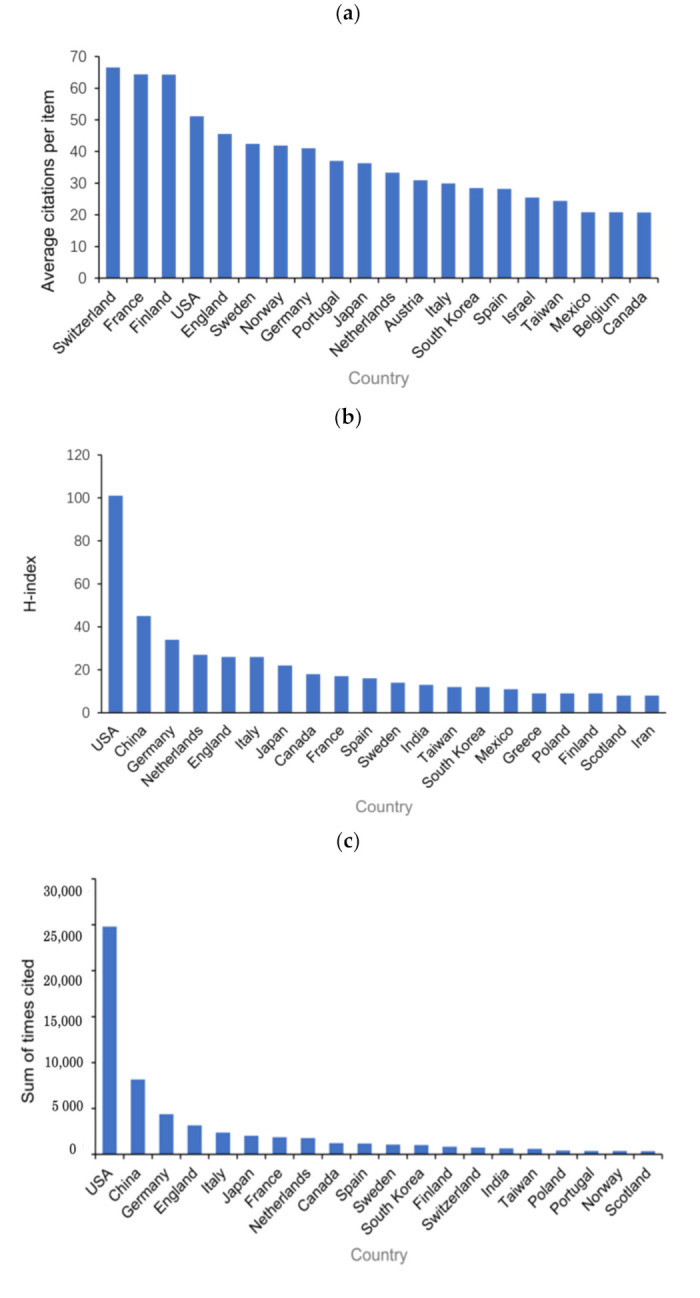
Citation frequency and H-index levels of different countries. (**a**) Total citations of the AS research articles from different countries. (**b**) Average citations per paper for articles from different countries. (**c**) The H-index of publications in the different countries.

United States publications were cited the most (24,795 times) with China ranking second (8151 times), followed by Germany (4366 times), England (3143 times), and Italy (2381 times) (Figure 2a). In terms of average citation frequency, Switzerland’s publications were cited the most (66.55 times). The second was France (64.38 times), followed by Finland (64.31 times), United States (51.12 times), and England (45.55 times) (Figure 2b).

### 3.3. H-Index

The h-index is an indicator of the scientific impact of an academic publishing career [28]. A country’s publications are ordered according to the number of times each was cited, from highest to lowest, and the list is scanned until the number of the last publication greater than or equal to the citation number is found [29]. Thus, the H-index is the number of papers published that were cited at least H times [30]. The H-index from United States authors was the highest at 101, followed by China (45), Germany (34), The Netherlands (27), and England (26) (Figure 2c).

### 3.4. Institutions and Publications

The contribution of institutions to a certain field is very important; the three most productive sources in the field were Washington U. (36, 2.13), Harvard U. (31, 1.83), and Karolinska Inst. (28, 1.65) (Table 1).

The journals with the largest number of publications were ‘Arteriosclerosis Thrombosis and Vascular Biology’ (62, 3.66), ‘Atherosclerosis’ (58, 3.43), and ‘Plos One’ (48, 2.84) (Table 2).

The publication “Oxidative Stress and Diabetic Complications” was cited 2370 times. It is the most frequently cited publication and has important guiding significance for the research in this field.

### 3.5. Bibliographic Coupling Analysis

**Figure 3:**
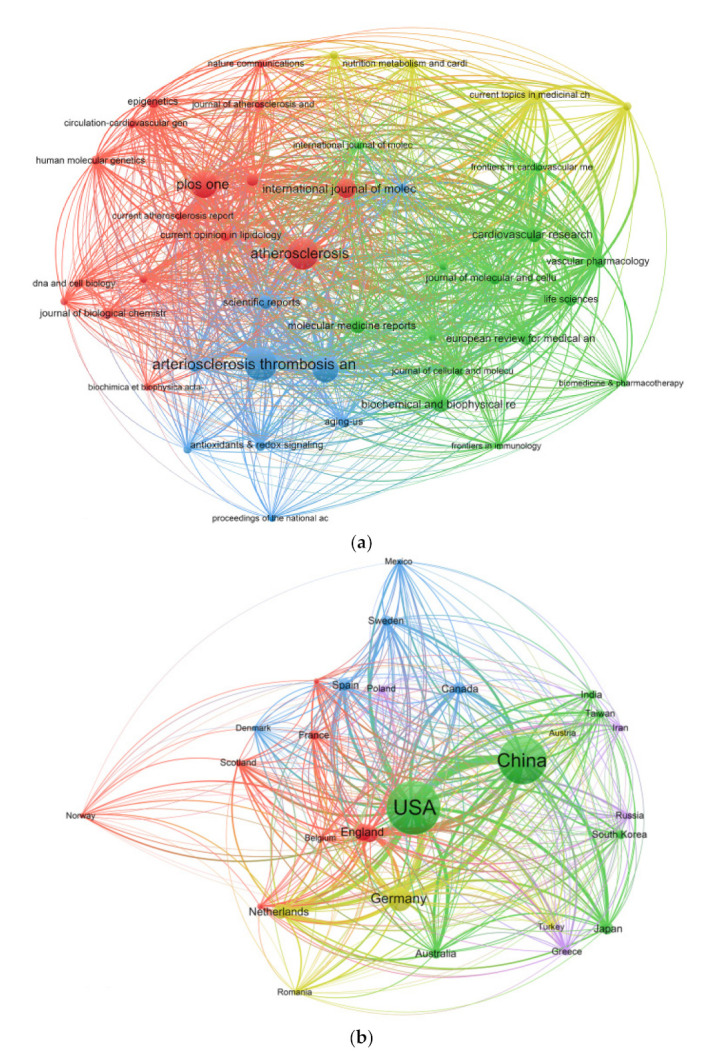

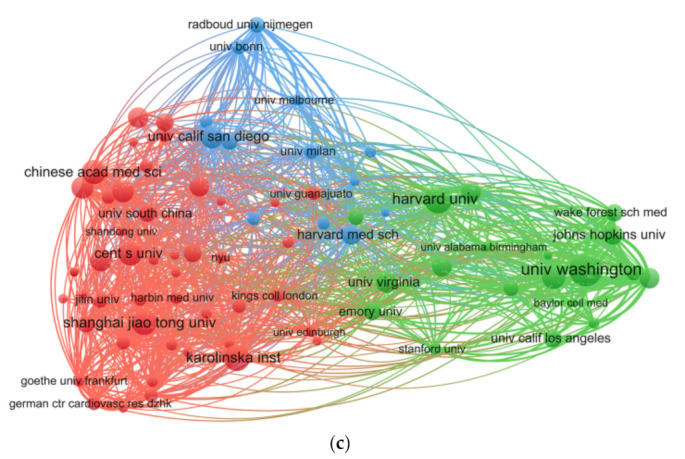
Bibliographic coupling analysis. (**a**) Coupling analysis chart of 48 journals with eight or more articles were analyzed. (**b**) Literature coupling analysis chart composed of 78 organizations with ten or more articles. (**c**) Literature coupling analysis chart of 29 countries with twelve or more articles. The line between two points in the figure shows that two journals, institutions, or countries had established a similarity relationship. The thicker the line, the closer the link between the two journals, institutions, or countries.

Literature coupling reflects the relationship between two bibliographies in terms of similarity of citations. Bibliographic coupling occurs when two papers cite the same reference in their respective bibliographies. This indicates that there may be internal relations and rules between two publications. The journal names of all publications were analyzed by VOSviewer. As can be seen in Figure 3a, there were 48 journals showing success through high total link strength (TLS). The five journals with the highest TLS were *Atherosclerosis* (9280), *Vascular Pharmacology* (6688), *Arteriosclerosis, Thrombosis, and Vascular Biology* (6636), *Circulation Research* (5519), and *the International Journal of Molecular Sciences* (5169).

The publications from 78 institutions were analyzed by VOSviewer and the minimum number of documents for inclusion of an organization was more than 10 (Figure 3b). The five universities with the highest TLS were Washington U. (14,991), Harvard (13,867), U. of Minnesota (13,448), Columbia U. (11,916), and Ludwig Maximilians U. (11,161).

The publications from 29 countries were analyzed by Vosviewer, and only those countries with at least 12 documents were included (Figure 3c). The five countries with the highest TLS were the United States (187,587), China (136,300), Germany (99,698), Italy (55,450), and The Netherlands (53,720).

### 3.6. Co-Occurrence Analysis

**Figure 4:**
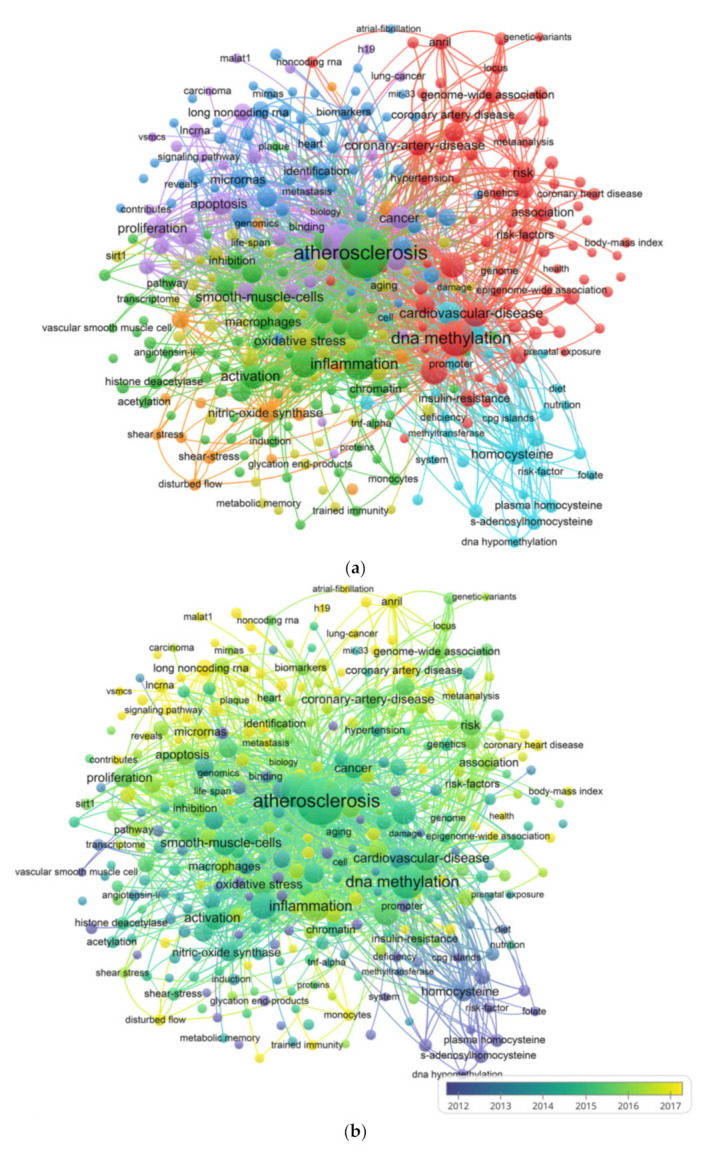
Co-occurrence analysis of global research about atherosclerosis. (**a**) Mapping of keywords in the research on atherosclerosis. The size of the points represents the frequency, and the keywords are divided into four clusters: molecular level research (left in blue), in vitro research (lower in yellow), in vivo research (right in red) and mechanism research (upper in green). (**b**) Distribution of keywords according to the mean frequency of appearance. Keywords in purple appeared earlier than those in green, and yellow keywords appeared later. The size of the points represents the citation frequency. A line between two points means that both were cited in one journal. A shorter line indicates a closer link between two journals. Points in the same color belong to the same research direction.

Co-occurrence analysis refers to the phenomenon that the same or different types of feature items appear together. The purpose of cooperative analysis is to determine popular areas and development directions for cooperative research. This has proved to be very important in the development of science monitoring systems and other disciplines. The title and abstract of the retrieved literature were opened through VOS Viewer software. The software associated the repeated keywords, set the frequency of keyword occurrence not less than 30 times, and generated interactive visual analysis images of the selected keywords through the software. As shown in Figure 4a, the 85 keywords were divided into four clusters: “molecular level research”, “mechanism research”, “in vitro research”, and “in vivo research”. These results are the most prominent topics in the epigenetic field of atherosclerosis. In the cluster of “molecular level research”, the main keywords were “atherosclerosis” and “expression”. In the “mechanism research” cluster, the main keywords were “DNA methylation” and “methylation”. In the “in vitro research” cluster, the main keywords were “smooth muscle cells” and “gene expression”. In the “in vivo research” cluster, the main keywords were “epigenetics” and “cardiovascular disease”. The VOS viewer colors keywords are based on the average number of times that they appear in all included publications. Purple means that keywords appear earlier, while yellow means that keywords appear later. Figure 4b shows that most of the studies before 2014 focused on “mechanism research” and “in vitro research”. However, based on trends in recent years, current research pays more attention to comprehensive development.

## 4. Discussion

Epigenetic processes are highly dynamic and reversible, and have specific ‘writers’ and ‘erasers’ that regulate gene expression. Targeting these epigenetic processes offers a great opportunity for discovery of new ways to prevent and treat atherosclerosis [14]. The VOSviewer supports the import and analysis of literature databases, general network data, and text data [28]. The core idea of the design of VOSviewer is ‘co-occurrence clustering’, which means that, if two things appear at the same time, they must be related [31]. There are many types of correlation, and their strength and direction are different. Different types of groups can be found by clustering based on the measurement index of relationship strength and direction [32]. Cluster analysis can reveal the research status of a field intuitively, and has great significance for guiding future research trends [33].

With the recognition that epigenetic modifications have a key role in atherosclerosis, the number of publications related to atherosclerosis in the epigenetic field began to increase every year from 1995 to 2020. This shows that atherosclerosis research has experienced rapid development in the field of epigenetics, and that epigenetic studies on atherosclerosis constitute a hot new area. In our study, we used bibliographic coupling analysis to establish similarity relationships between publications in different countries, journals, and institutions. These data indicated that *Arteriosclerosis Thrombosis and Vascular Biology* was the most closely related journal. The key words in the titles and abstracts of the included studies were analyzed to yield a map showing the collaborative network [34]. From these results, the research hotspots and future development trends in this field were identified and predicted [35]. Based on the co-occurrence network, we identified four potential research trends: molecular level research, mechanism research, in vitro research, and in vivo research. Our study clarified the association between previous investigations and future research trends.

The visualization diagram is similar to the co-occurrence diagram, but the items are colored differently [30]. This effective method is used to monitor research progress [29]. For example, the overlay visualization shows that the study of DNA methylation in atherosclerosis is a hot topic in this field. Moreover, according to our research results, there have been significantly more studies on lncRNA in the field of atherosclerosis since 2017. We speculate that research focused in this direction will become a hotspot in epigenetic studies of atherosclerosis.

Our studies have evaluated the trends and current status of atherosclerosis research in the area of epigenetics through bibliometric and visual analysis, the results of which also have some limitations. Although we also searched the keywords related to epigenetic modification repeatedly, we still could not avoid missing relevant articles. In addition, some new, high-quality publications may not receive attention because of their low citation frequency. These problems are the limitations of this article. Researchers need to pay greater attention to the latest published research in related fields, in order to more accurately predict the most important research trends in this field.

## 5. Conclusions

Although considerable evidence has accumulated demonstrating how epigenetic modification plays an important role in atherosclerosis, more specific research still needs to be explored by scientists. The results of this study summarized the research status of epigenetic modifications in atherosclerosis, and predicted the future research trends in this field. An in-depth study of this field will ensure that we have a more accurate understanding of atherosclerotic disease and provide clear directions for clinical research. The bibliometric data show that atherosclerosis is developing rapidly with the study of DNA methylation, which is currently a hot research area that should be taking its place alongside the previous emphasis on lncRNA studies in atherosclerosis in the field of epigenetics.

## Figures and Tables

**Table 1:** Most productive institutions in the field of atherosclerosis.

Institution	No. (%) of Publications (*N* = 1848)
Washington University	36 (1.95)
Harvard University	33 (1.79)
Karolinska inst	28 (1.52)
Shanghai jiaotong University	28 (1.52)
Chinese Academy of Medical Sciences	27 (1.46)

**Table 2:** The top five publications.

Rank	Journal	No. (%) of Publications (*N* = 1848)
1	Arteriosclerosis thrombosis and vascular biology	66(3.57)
2	Atherosclerosis	61(3.30)
3	Circulation research	50(2.71)
4	Plos one	49(2.65)
5	International journal of molecular	33(1.79)

## Data Availability

Search keywords, collect literature and use websites: https://www.webofscience.com/wos/alldb/basic-search.

## References

[1] Hansson G.K., Hermansson A. (2011). The immune system in atherosclerosis. Nat. Immunol..

[2] Cavalli G., Heard E. (2019). Advances in epigenetics link genetics to the environment and disease. Nature.

[3] Koelwyn G.J., Corr E.M., Erbay E., Moore K.J. (2018). Regulation of macrophage immunometabolism in atherosclerosis. Nat. Immunol..

[4] Kuznetsova T., Prange K.H.M., Glass C.K., de Winther M.P.J. (2020). Transcriptional and epigenetic regulation of macrophages in atherosclerosis. Nat. Rev. Cardiol..

[5] Karthika C.L., Ahalya S., Radhakrishnan N., Kartha C.C., Sumi S. (2020). Hemodynamics mediated epigenetic regulators in the pathogenesis of vascular diseases. Mol. Cell Biochem..

[6] Kwon D.H., Ryu J., Kim Y.K., Kook H. (2020). Roles of Histone Acetylation Modifiers and Other Epigenetic Regulators in Vascular Calcification. Int. J. Mol. Sci..

[7] Napoli C., Benincasa G., Schiano C., Salvatore M. (2020). Differential epigenetic factors in the prediction of cardiovascular risk in diabetic patients. Eur. Heart J. Cardiovasc. Pharmacother..

[8] Mason R.J., Vondriska T.M. (2019). Chromatin Is the Same in a Relative Way (But You’re Older). Circ. Res..

[9] Scisciola L., Rizzo M.R., Cataldo V., Fontanella R.A., Balestrieri M.L., D’Onofrio N., Marfella R., Paolisso G., Barbieri M. (2020). Incretin drugs effect on epigenetic machinery: New potential therapeutic implications in preventing vascular diabetic complications. FASEB J..

[10] Toma L., Sanda G.M., Niculescu L.S., Deleanu M., Sima A.V., Stancu C.S. (2020). Phenolic Compounds Exerting Lipid-Regulatory, Anti-Inflammatory and Epigenetic Effects as Complementary Treatments in Cardiovascular Diseases. Biomolecules.

[11] Gorabi A.M., Penson P.E., Banach M., Motallebnezhad M., Jamialahmadi T., Sahebkar A. (2020). Epigenetic control of atherosclerosis via DNA methylation: A new therapeutic target?. Life Sci..

[12] Liu M., Yan M., Lv H., Wang B., Lv X., Zhang H., Xiang S., Du J., Liu T., Tian Y. (2020). Macrophage K63-Linked Ubiquitination of YAP Promotes Its Nuclear Localization and Exacerbates Atherosclerosis. Cell Rep..

[13] Pirillo A., Svecla M., Catapano A.L., Holleboom A.G., Norata G.D. (2021). Impact of protein glycosylation on lipoprotein metabolism and atherosclerosis. Cardiovasc. Res..

[14] Xu S., Pelisek J., Jin Z.G. (2018). Atherosclerosis Is an Epigenetic Disease. Trends Endocrinol. Metab..

[15] Gillette T.G., Hill J.A. (2015). Readers, writers, and erasers: Chromatin as the whiteboard of heart disease. Circ. Res..

[16] Xu S., Kamato D., Little P.J., Nakagawa S., Pelisek J., Jin Z.G. (2019). Targeting epigenetics and non-coding RNAs in atherosclerosis: From mechanisms to therapeutics. Pharmacol. Ther..

[17] Lu Y., Sun Y., Drummer C.t., Nanayakkara G.K., Shao Y., Saaoud F., Johnson C., Zhang R., Yu D., Li X. (2019). Increased acetylation of H3K14 in the genomic regions that encode trained immunity enzymes in lysophosphatidylcholine-activated human aortic endothelial cells—Novel qualification markers for chronic disease risk factors and conditional DAMPs. Redox Biol..

[18] Jiang X., Liu Z., Qi X. (2021). LncRNA BANCR induced vascular smooth muscle cell proliferation by downregulating miR-34c methylation in atherosclerosis. J. Thromb. Thrombolysis.

[19] Li Y., Xu S., Mihaylova M.M., Zheng B., Hou X., Jiang B., Park O., Luo Z., Lefai E., Shyy J.Y. (2011). AMPK phosphorylates and inhibits SREBP activity to attenuate hepatic steatosis and atherosclerosis in diet-induced insulin-resistant mice. Cell Metab..

[20] Zhang Y., Qian H., Wu B., You S., Wu S., Lu S., Wang P., Cao L., Zhang N., Sun Y. (2020). E3 Ubiquitin ligase NEDD4 familyregulatory network in cardiovascular disease. Int. J. Biol. Sci..

[21] Menni C., Gudelj I., Macdonald-Dunlop E., Mangino M., Zierer J., Besic E., Joshi P.K., Trbojevic-Akmacic I., Chowienczyk P.J., Spector T.D. (2018). Glycosylation Profile of Immunoglobulin G Is Cross-Sectionally Associated with Cardiovascular Disease Risk Score and Subclinical Atherosclerosis in Two Independent Cohorts. Circ. Res..

[22] Danthi N., Wu C.O., Shi P., Lauer M. (2014). Percentile ranking and citation impact of a large cohort of National Heart, Lung, and Blood Institute-funded cardiovascular R01 grants. Circ. Res..

[23] Shi B., Wei W., Qin X., Zhao F., Duan Y., Sun W., Li D., Cao Y. (2019). Mapping theme trends and knowledge structure on adipose-derived stem cells: A bibliometric analysis from 2003 to 2017. Regen. Med..

[24] Zhang X., Zhao X., Liu K., Che Y., Qiu X., Qu Y., Sun X., Song J. (2020). Bufalin: A Systematic Review of Research Hotspots and Antitumor Mechanisms by Text Mining and Bioinformatics. Am. J. Chin. Med..

[25] Wang B., Xing D., Zhu Y., Dong S., Zhao B. (2019). The State of Exosomes Research: A Global Visualized Analysis. BioMed Res. Int..

[26] Shukla N., Merigó J.M., Lammers T., Miranda L. (2020). Half a century of computer methods and programs in biomedicine: A bibliometric analysis from 1970 to 2017. Comput. Methods Programs Biomed..

[27] Polero P., Rebollo-Seco C., Adsuar J.C., Pérez-Gómez J., Rojo-Ramos J., Manzano-Redondo F., Garcia-Gordillo M., Carlos-Vivas J. (2020). Physical Activity Recommendations during COVID-19: Narrative Review. Int. J. Environ. Res. Public Health.

[28] Manyangu G., Dineen B., Geoghegan R., Flaherty G. (2019). Descriptive bibliometric analysis of global publications in lifestyle-based preventive cardiology. Eur. J. Prev. Cardiol..

[29] Bertoli-Barsotti L., Lando T. (2017). A theoretical model of the relationship between the h-index and other simple citation indicators. Scientometrics.

[30] Adnan S., Ullah R. (2018). Top-cited Articles in Regenerative Endodontics: A Bibliometric Analysis. J. Endod..

[31] Gao J., Xing D., Dong S., Lin J. (2020). The primary total knee arthroplasty: A global analysis. J. Orthop. Surg. Res..

[32] Mao X., Guo L., Fu P., Xiang C. (2020). The status and trends of coronavirus research: A global bibliometric and visualized analysis. Medicine.

[33] Yin M.C., Wang H.S., Yang X., Xu C.Q., Wang T., Yan Y.J., Fan Z.X., Ma J.M., Ye J., Mo W. (2020). A Bibliometric Analysis and Visualization of Current Research Trends in Chinese Medicine for Osteosarcoma. Chin. J. Integr. Med..

[34] Xia N., Xie Q., Griffin M.A., Ye G., Yuan J. (2020). Antecedents of safety behavior in construction: A literature review and an integrated conceptual framework. Accid. Anal. Prev..

[35] Bougioukas K.I., Vounzoulaki E., Mantsiou C.D., Savvides E.D., Karakosta C., Diakonidis T., Tsapas A., Haidich A.B. (2020). Methods for depicting overlap in overviews of systematic reviews: An introduction to static tabular and graphical displays. J. Clin. Epidemiol..

